# Does the “learning effect” caused by digital devices exaggerate sports visual training outcomes? A systematic review and meta-analysis

**DOI:** 10.3389/fphys.2025.1664572

**Published:** 2025-09-05

**Authors:** Yuqiang Guo, Tinggang Yuan, Mulin Yang, Jinyu Qiu

**Affiliations:** ^1^ Science Research Center of Sports Training, China Institute of Sport Science, Beijing, China; ^2^ School of Physical Education, Shanghai University of Sport, Shanghai, China; ^3^ Physical Fitness Training Research Center, China Institute of Sport Science, Beijing, China; ^4^ Xinxin School, The Affiliated High School of Peking University, Beijing, China

**Keywords:** digital-based training, visual-cognitive skills, practice effect, task similarity, sports vision training

## Abstract

**Objective:**

Digital-based visual training (VT) is widely employed to improve visual-cognitive performance, yet its efficacy may be confounded by the “learning effect”.

**Methods:**

A systematic literature search was conducted across PubMed, Web of Science, MEDLINE, SPORTDiscus, and Cochrane Library, covering all studies published up to 8 May 2025. The search was limited to peer-reviewed articles written in English. Only randomized controlled trials (RCTs) that included both baseline and post-intervention measures of visual-cognitive performance were eligible. Subgroup analysis was conducted based on the presence or absence of task similarity between training and testing conditions, to assess potential bias introduced by the “learning effect”.

**Results:**

The search identified 3,798 articles, of which 33 RCTs involving 1,048 participants met the inclusion criteria for meta-analysis. VT was found to significantly improve visual attention, reaction time, decision-making time, decision-making accuracy, and eye–hand coordination. Subgroup analyses revealed that studies classified as “learning effect present” (LE+) consistently reported substantially larger effect sizes than those without (LE−). Significant between-group differences were observed for visual attention (SMD = 1.65 vs. 0.07; *p* = 0.00), reaction time (SMD = 2.66 vs. 0.50; *p* = 0.00), and decision-making accuracy (SMD = 1.46 vs. 0.62; *p* = 0.03), indicating that task similarity may artificially inflate performance outcomes.

**Conclusion:**

These findings indicate that observed improvements may reflect task familiarity rather than true cognitive enhancement. To improve evaluation validity, future studies should avoid task redundancy, incorporate retention testing, and adopt structurally distinct outcome measures.

## 1 Introduction

Sports vision refers to the integrated skills to perceive, process, and respond to critical environmental information in competitive scenarios (Erickson, 2021). It not only serves as a vital bridge between decision-making and motor execution but also directly impacts athletic performance under high-speed confrontations, tactical adaptations, and extreme time pressure (Erickson et al., 2011). Enhancing sports vision has become a major focus of research and practice in elite athletic training (Appelbaum and Erickson, 2018; Laby and Appelbaum, 2021). The integration of sports science and digital technology has led to the widespread adoption of diverse visual training (VT) methods in elite sports, aiming to improve athletes’ visual function and optimize information processing and decision-making under pressure (Kittel et al., 2024; Jothi et al., 2025). Empirical studies and systematic reviews have shown that digital VT methods—such as stroboscopic visual training (Jothi et al., 2025; Zwierko et al., 2023; Zwierko et al., 2024), perceptual-cognitive training (Müller et al., 2024; Kassem et al., 2024; Zhu et al., 2024), and virtual reality training (Liu et al., 2024; Skopek et al., 2023) — can significantly improve key visual-cognitive skills like attention, reaction time, and decision-making, demonstrating strong potential for practical implementation. However, Fransen (Fransen, 2024) argued that current scientific evidence is insufficient to support the “far transfer” of perceptual or cognitive training to athletic performance. Many commercial digital training tools appear to facilitate “near transfer” but fail to improve on-field performance (Harris et al., 2018). This discrepancy may result from structural similarities between training and testing tasks, leading to a so-called “learning effect” (Basner et al., 2020). The term “learning effect” denotes performance improvements driven by procedural familiarity with tasks or devices rather than genuine skill acquisition. In such cases, repeated exposure enhances test scores through familiarity alone, independent of true training-induced adaptation.

Recent studies (Basner et al., 2020; Chaloupka and Zeithamova, 2024) have highlighted that the structural overlap between training and testing tasks—a common feature in cognitive training research—can trigger a “learning effect”, whereby participants improve on post-tests not due to true skill enhancement, but because of familiarity with stimuli, response formats, or device interfaces. If not adequately controlled, this bias may result in improvements driven by faster procedural memory or task-specific strategy optimization, rather than genuine gains in visual-cognitive skills. Similar issues are evident in VT research. Krasich, Ramger (Krasich et al., 2016) reported that repeated testing with digital devices led to linear performance improvements over a short period, largely due to growing familiarity with the equipment. Reported high training effects in this field may not truly reflect visual system plasticity, but may instead overestimate efficacy due to “learning effect”. Meta-analyses by Müller, Morris-Binelli (Müller et al., 2024) and Zhu, Zheng (Zhu et al., 2024) found that improvements in decision-making through perceptual-cognitive training were greater in laboratory tests than in field-based assessments (SMD = 1.26 vs. 0.85; 1.51 vs. 0.65), highlighting insufficient “far transfer” effects. To date, no study has systematically examined the “learning effect” as a moderator of VT outcomes. This gap represents an important methodological hindrance, as task-related familiarity may artificially inflate post-test performance and mask the true efficacy of training interventions. Consequently, the bias introduced by learning effects has persisted largely unaddressed in the literature.

Therefore, based on the above research background, this study conducted a systematic review and meta-analysis to examine whether the “learning effect” moderates the outcomes of VT interventions. Subgroup analyses were employed to compare studies with and without the presence of “learning effect”, aiming to identify a potential source of bias that may have been overlooked in previous research. The findings are intended to provide methodological guidance and empirical evidence for future studies in the areas of intervention design, outcome measure selection, and interpretative frameworks.

## 2 Materials and methods

This systematic review and meta-analysis followed the PRISMA (Preferred Reporting Items for Systematic Reviews and Meta-Analyses) guidelines (Moher et al., 2010) and was preregistered on PROSPERO (ID: CRD420251020142).

### 2.1 Search strategy

**FIGURE 1:**
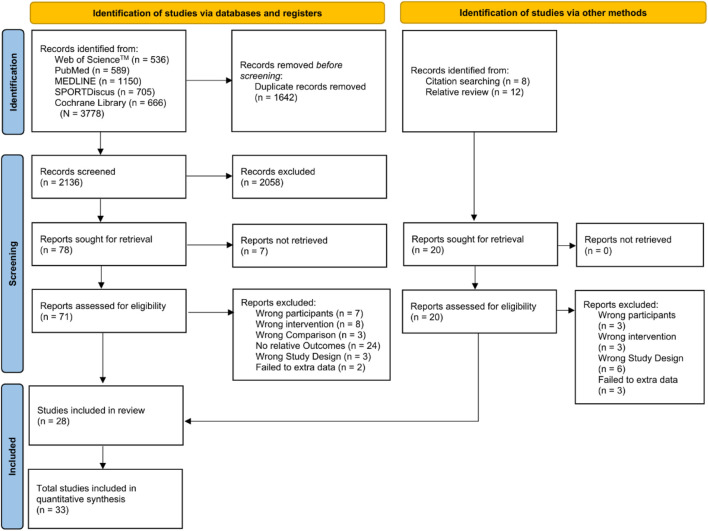
PRISMA flow diagram for study inclusion.

A comprehensive literature search up to 8 May 2025 was conducted across five electronic databases: PubMed, Web of Science (Core Collection), MEDLINE, SPORTDiscus, and Cochrane Library. Boolean search operators (“AND”, “OR”) were applied with combinations of the following keywords: “visual training”, “vision training”, “eye training”, “visuomotor training”, “visual motor training”, “perceptual training”, “perceptual-cognitive training”, “temporal occlusion training”, “strobe training”, “stroboscopic training”, “virtual reality training”, “VR training”, “visual-spatial training”, “visual search training”, “multiple object tracking training”, “randomized controlled trial”, “random allocation”, “RCT”, “randomized” and “randomly”. The full search strategy is provided in Supplementary Appendix 1. Manual searches of reference lists from included studies were conducted. In addition, narrative and systematic reviews (Jothi et al., 2025; Müller et al., 2024; Zhu et al., 2024; Lochhead et al., 2024) on related topics were retrieved. Automated duplicate detection and title-abstract screening were performed using Rayyan software (Ouzzani et al., 2016). After all duplicates were removed, two reviewers (YG and JQ) independently assessed the identified publications using predetermined criteria. Any disagreements were resolved by consultation with a third reviewer (MY). When the titles and abstracts suggested that the article might meet the inclusion criteria, full-text articles were retrieved. If a manuscript was unavailable, the corresponding author was contacted by email. The study selection process is illustrated in Figure 1.

### 2.2 Inclusion and exclusion criteria

Inclusion and exclusion criteria were established using a revised PICOS framework (Amir-Behghadami and Janati, 2020). Only English-language randomized controlled trials published in peer-reviewed journals were included; studies in other languages or those that were non-randomized, uncontrolled, or cross-sectional were excluded.

#### 2.2.1 Types of population

Participants included in the study were not restricted by gender but were required to have a certain level of sport experience and engagement in a specific sport discipline. According to the criteria (McKay et al., 2022), all participants were classified at least at Tier 2 (Trained) or above, as individuals at this level possess a relatively stable foundation in physical fitness, technical skills, and sport-specific performance. This ensures greater reliability and validity of performance-related data and minimizes measurement error and bias associated with low physical activity levels. Additionally, participants were required to be older than 10 years (Sánchez-González et al., 2022; Leat et al., 2009) and younger than 60 years (Mehta, 2015), in order to avoid the confounding effects of growth, development, and age-related decline on visual and motor functions. All participants had to be healthy individuals with no existing musculoskeletal injuries (e.g., chronic ankle instability) or visual impairments (e.g., high myopia) that could influence the outcomes of visual ability assessments.

#### 2.2.2 Types of intervention

According to a recent review (Lochhead et al., 2024), VT should be defined as a structured, task-specific intervention aimed at enhancing visual-perceptual and visual-cognitive skills that are critical to athletic performance. Based on the intended mechanisms (Appelbaum and Erickson, 2018), digitally-based VT can be classified into three categories: Component Skill Training, Naturalistic Training Approaches, and Integrated Training Batteries. Therefore, included studies must align with the core characteristics of these training modalities. In addition, acute intervention studies were excluded; thus, only interventions with a minimum duration of 1 week were included. This threshold was applied to exclude acute or single-session studies, which primarily capture immediate practice effects rather than training-based adaptations (Appelbaum and Erickson, 2018; Smith and Mitroff, 2012).

#### 2.2.3 Types of comparison

In this review, single-arm trials or two-armed VT intervention design studies without a valid comparator were excluded. Control groups may include either active controls (e.g., alternative training such as regular training or training without visual intervention condition) or passive controls (no intervention). If a study incorporates both active and passive control (no-intervention) conditions, the passive control group were prioritized, because they minimize confounding effects from alternative training programs and provide a clearer estimate of the true efficacy of VT.

#### 2.2.4 Types of outcomes

The visual skills measures were categorized into two main domains: visual-perceptual and visual-cognitive skills. According to the study by Krasich, Ramger (Krasich et al., 2016), significant learning effects were observed in tasks with high visuomotor control demands (Perception Span, Hand Reaction Time, Go/No Go, and Eye-Hand Coordination), whereas no significant progress was seen in tasks involving only visual sensitivity—measures that are also difficult to improve through specific VT (Shekar et al., 2021). This suggests that the observed improvements in performance due to repeated testing with digital devices were primarily attributable to participants’ increased familiarity with the equipment rather than the intervention itself. Therefore, the indicators included in the present meta-analysis were primarily visual-cognitive skills, including visual attention, reaction time, decision-making skills, and eye hand coordination. Studies that did not use digital devices to assess these specific outcomes were excluded from the meta-analysis. Digital devices were defined as electronic or computerized tools that provide standardized visual stimuli and/or automatically record responses, ensuring objective and reproducible measurement. Eligible devices included video-based testing platforms, multiple-object tracking software, light-board systems, and virtual reality headsets. By contrast, studies relying solely on in-game performance indicators (e.g., passing or shooting accuracy) or subjective coach observation without digital instrumentation were excluded. To allow for the calculation of effect sizes (ES), studies were required to provide adequate statistical information, including pre-post repeated measures and/or change scores along with their corresponding standard deviations. Studies were not excluded based on the specific methodologies employed to assess these outcomes.

#### 2.2.5 Types of study design

Only randomized controlled trials (RCTs) were included.

### 2.3 Data extraction procedures

Two reviewers (YG and JQ) independently extracted the data using a customized Excel worksheet (Microsoft Corp., Redmond, WA, United States). Any discrepancies during the extraction process were resolved through discussion, with arbitration by a third reviewer (MY) when consensus could not be reached. The following data were extracted from each included study: (1) authors and year of publication; (2) participant characteristics, including sample size, sex, age, sport type, and performance level; (3) intervention characteristics, such as training modality, frequency, and duration; and (4) outcome measures, including the test instruments used, as well as the reported means, standard deviations, and standard errors for both intervention and control groups. In accordance with the approach proposed by Thiele, Prieske (Thiele et al., 2020), when multiple outcomes were reported, the outcome with the most significant was prioritized. For studies lacking complete numerical data or reporting results only in graphical form, the original authors were contacted to obtain the necessary information. If the data could not be retrieved through author correspondence, values were estimated from figures using WebPlotDigitizer website (https://automeris.io/WebPlotDigitizer) (Burda et al., 2017).

### 2.4 Risk of bias

The risk of bias and methodological quality of the included studies were independently evaluated by two reviewers (YG and JQ), following the Cochrane Risk of Bias 2.0 framework (Burda et al., 2017). This tool assesses potential bias across several domains, including: random sequence generation, allocation concealment, blinding of participants and personnel, blinding of outcome assessors, completeness of outcome data, and selective outcome reporting. Each domain was rated as having a low risk, high risk, or some concerns. Any discrepancies between the two reviewers were resolved through consensus discussions, with arbitration by a third reviewer (MY) when necessary.

### 2.5 Statistical analysis

#### 2.5.1 Data synthesis and effect measures

To evaluate the effectiveness of VT on visual-cognitive skills and to investigate whether the “learning effect” has influenced the outcomes of existing studies, the present meta-analysis was conducted following the procedures outlined below. Following data extraction based on the aforementioned procedures, the first step involved calculating the mean difference (*MDdiff*) and the corresponding standard deviation (*SD*_*diff*_). The *MD*_*diff*_ of the intervention and control groups between the pre- and post-test changes was calculated using Equation 1. The standard deviation (*SD*_*diff*_) of the changes was determined using Equation 2. In cases where the correlation coefficient (*Corr*) was not explicitly reported in the studies, it was calculated through correlation analysis based on raw data. If the original data could not be obtained, the original research teams were contacted for provision. If these methods were not feasible, *Corr* was assumed to be 0.5, as suggested by the Cochrane Handbook (Cumpston et al., 2019). This intermediate value balances the potential under- and over-estimation of variability in the absence of study-specific correlation data.

$$MD_{diff}=M_{post}‐M_{pre}$$ (1)

$${SD}_{diff}=\sqrt{{SD_{pre}}^{2}+{SD_{post}}^{2}‐2\times Corr\times SD_{pre}\times SD_{post}}$$ (2)

In accordance with Hedges and Olkin (Hedges and Olkin, 1985), the standardized mean differences (SMD) were adjusted for sample size using the correction factor 1-[3/(4 N-9)]. Given that the sample sizes of most of the included studies are small, to enhance the reliability of the research, Hedge’s g, which has been adjusted for bias and based on Equations 3, 4, was used as the effect size indicator for each study.

$${\text{Hedge}}^{\prime }s\;g=\left(\frac{\text{VT}\left(M_{\text{change}}\right)‐\text{CON}\left(M_{\text{change}}\right)}{SD_{pooled}}\right)\times \left(1‐\frac{3}{4\left(n_{1}+n_{2}\right)‐9}\right)$$ (3)

$${\text{SD}}_{pooled}=\sqrt{\frac{\left(\left(n_{1}‐1\right)\times {\text{SD}}_{1}^{2}+\left(n_{2}‐1\right)\times {\text{SD}}_{2}^{2}\right)}{\left(n_{1}+n_{2}‐2\right)}}$$ (4)

In the above formula, M_change_ represents the mean change from pre-to post-intervention in the VT and control groups, respectively. SD_*pooled*_ denotes the pooled standard deviation of the change scores across both groups, while *n*_1_ and *n*_2_, as well as SD_1_ and SD_2_, refer to the sample sizes and standard deviations of the two groups, respectively. Hedge’s *g* values were classified as small (<0.50), medium (0.50–0.80), and large (≥0.80) (Hedges and Olkin, 1985).

#### 2.5.2 Meta-analysis and test for heterogeneity

The meta-analysis and data visualization were conducted using the “meta” and “metafor” packages in R software (version 4.3.3, R Core Team, Vienna, Austria). A conventional two-level meta-analysis approach was applied, utilizing the inverse-variance weighting method. Effect sizes were synthesized under a random-effects model based on the DerSimonian–Laird method (DerSimonian and Laird, 1986). This model assumes that effect sizes are drawn from a distribution of true effects rather than from a single homogeneous population (Cumpston et al., 2019). By incorporating between-study variability, it allows for a more generalizable and accurate estimation of the overall effect size. To avoid unit-of-analysis errors, when multiple intervention groups were compared against a shared control group, the sample size of the shared group was evenly divided across comparisons (Poon et al., 2024).

Between-study heterogeneity was evaluated using both the *I* ^2^ statistic and Cochran’s Q (Chi-square) test. The degree of heterogeneity, as indicated by *I* ^2^, was categorized as low (<25%), moderate (25%–50%), high (50%–75%), or considerable (≥75%) in accordance with established guidelines (Higgins et al., 2003). These metrics provided insight into the extent to which variability in effect sizes was attributable to true heterogeneity rather than sampling error.

#### 2.5.3 Subgroup analysis

To explore whether the presence of “learning effect” moderated the observed VT outcomes, a subgroup analysis was performed. A systematic evaluation of the full texts was conducted, focusing on the consistency between the training tasks and the outcome assessments, including the devices used. Studies were classified into the Learning Effect Present (LE+) group if both of the following criteria were met: (1) the digital device used for training and testing was identical or highly similar (Poltavski et al., 2021), and (2) the structure and mode of the training task closely matched those of the outcome measure (Fransen, 2024). Studies that did not meet both criteria—or met only one—were assigned to the Learning Effect Absent (LE−) group. For example, studies were classified as “LE+” if the intervention involved a multiple-object tracking task using the same or a highly similar digital platform as the outcome test, or if a computerized visual reaction-time training program was evaluated with the same reaction-time software during testing. Differences in pooled effect sizes between subgroups were tested using a mixed-effects model (Christ, 2009). When the number of studies meeting the inclusion criteria (n = 5) (Deeks et al., 2019) is insufficient to perform a subgroup analysis, a systematic review were conducted for that outcome.

#### 2.5.4 Risk of publication bias and sensitivity analysis

To evaluate the presence of publication bias, contour-enhanced funnel plots (Peters et al., 2008) were generated and Egger’s test (Fernández-Castilla et al., 2021) was performed, provided that the number of included studies in the respective analysis was ten or more. A p-value greater than 0.05 was interpreted as indicating no significant risk of publication bias. These methods allow for both visual and statistical assessment of asymmetry in the distribution of effect sizes, thereby assisting in determining the robustness and reliability of the pooled estimates. A leave-one-out sensitivity analysis was conducted by sequentially excluding each individual study to assess whether the overall pooled effect size was disproportionately influenced by any single study.

## 3 Results

### 3.1 Study characteristics

A total of 3,798 studies were retrieved from PubMed (n = 589), Web of Science™ (n = 536), MEDLINE (n = 1,150), SPORTDiscus (n = 705), and Cochrane Library (n = 666). Additionally, 20 records were identified through manual search. After removing duplicates and applying predefined inclusion and exclusion criteria, 33 studies (Gabbett et al., 2007; Maman et al., 2011; Paul et al., 2011; Serpell et al., 2011; Schwab and Memmert, 2012; Lorains et al., 2013; Murgia et al., 2014; Nimmerichter et al., 2015; Alder et al., 2016; Alsharji and Wade, 2016; Hohmann et al., 2016; Milazzo et al., 2016; Romeas et al., 2016; Gray, 2017; Brenton et al., 2019a; Brenton et al., 2019b; Petri et al., 2019; Romeas et al., 2019; Liu et al., 2020; Schumac et al., 2020; Bidil et al., 2021; Ehmann et al., 2022; Harenberg et al., 2022; Theofilou et al., 2022; Fortes et al., 2023; Phillips et al., 2023; Zwierko et al., 2023; Di et al., 2024; Guo et al., 2024; Lachowicz et al., 2024; Lucia et al., 2024; Mancini et al., 2024; Rodrigues et al., 2025) were included in the meta-analysis (Figure 1).

All included studies adopted a randomized controlled trial design, involving a total of 1,048 participants. The participants were athletes from a variety of sports, including soccer (Lorains et al., 2013; Murgia et al., 2014; Nimmerichter et al., 2015; Romeas et al., 2016; Schumac et al., 2020; Ehmann et al., 2022; Harenberg et al., 2022; Theofilou et al., 2022; Fortes et al., 2023; Phillips et al., 2023; Rodrigues et al., 2025), volleyball (Zwierko et al., 2023; Mancini et al., 2024), softball (Gabbett et al., 2007), tennis (Maman et al., 2011), table tennis (Paul et al., 2011), rugby (Serpell et al., 2011), hockey (Schwab and Memmert, 2012), badminton (Alder et al., 2016; Romeas et al., 2019; Bidil et al., 2021), handball (Alsharji and Wade, 2016; Hohmann et al., 2016), karate (Milazzo et al., 2016; Petri et al., 2019), baseball (Gray, 2017; Liu et al., 2020), cricket (Brenton et al., 2019a; Brenton et al., 2019b), fencing (Di et al., 2024), skeet shooting (Guo et al., 2024), esports (Lachowicz et al., 2024) and basketball (Lucia et al., 2024). The sample sizes of individual studies ranged from 15 to 80, with intervention durations spanning 1 week to 6 months. Training frequency varied between one and seven sessions per week, and each session lasted from 6 to 180 min.

The VT interventions were classified into the following categories: (1) perceptual-cognitive training [*n* = 10 studies (Gabbett et al., 2007; Serpell et al., 2011; Lorains et al., 2013; Murgia et al., 2014; Nimmerichter et al., 2015; Alder et al., 2016; Alsharji and Wade, 2016; Hohmann et al., 2016; Brenton et al., 2019a; Schumac et al., 2020)]; (2) visuomotor coordination training [*n* = 13 studies (Maman et al., 2011; Paul et al., 2011; Schwab and Memmert, 2012; Brenton et al., 2019a; Brenton et al., 2019b; Liu et al., 2020; Bidil et al., 2021; Theofilou et al., 2022; Di et al., 2024; Guo et al., 2024; Lucia et al., 2024; Mancini et al., 2024; Rodrigues et al., 2025)]; (3) multiple object tracking training [*n* = 4 studies (Romeas et al., 2016; Romeas et al., 2019; Ehmann et al., 2022; Phillips et al., 2023)]; (Laby and Appelbaum, 2021); stroboscopic visual training [*n* = 3 studies (Liu et al., 2020; Fortes et al., 2023; Zwierko et al., 2023)]; (Kittel et al., 2024); virtual reality training [*n* = 3 studies (Gray, 2017; Petri et al., 2019; Lachowicz et al., 2024)]. Furthermore, upon detailed evaluation of the methodological rigor across studies, only eleven were ultimately considered to report outcome measures that were not confounded by potential “learning effect”. Further details regarding study characteristics and intervention protocols are summarized in Supplementary Appendix Table 2.1.

### 3.2 Risk of bias

The risk of bias was evaluated using the Cochrane Risk of Bias 2.0 (RoB 2) tool, and the results are presented in Supplementary Appendix Figures 3.1 and 3.2. Although all included studies identified themselves as randomized controlled trials, only 11 clearly described the method used for random sequence generation. Consequently, the domain D1 (randomization process) was rated as “low risk” in these studies, while the others were judged as having “some concerns” due to insufficient reporting on randomization procedures. For D3 (missing outcome data), six studies were classified as “high risk” owing to substantial attrition that resulted in marked imbalance between groups. In terms of D4 (measurement of the outcome), most studies (n = 23) were rated as having “high risk” due to employing subjective evaluation methods and the presence of “learning effect”. In addition, nine other studies were judged as having “some concerns” due to the lack of blinding in outcome assessment. All included studies were marked as having “some concerns” for D5 (selection of the reported result), primarily due to incomplete reporting or absence of pre-specified analysis plans. Overall, 22 studies were deemed to be at “high risk”, and the remaining were categorized as having “some concerns”. It should be noted that the high proportion of studies rated as “high risk” was primarily driven by Domain 4, where the presence of “learning effects” compromised the validity of outcome measures. In addition, several studies suffered from high attrition rates. These limitations may have inflated the reported effects and should be considered when interpreting the overall findings.

### 3.3 Main analyses

**FIGURE 2:**
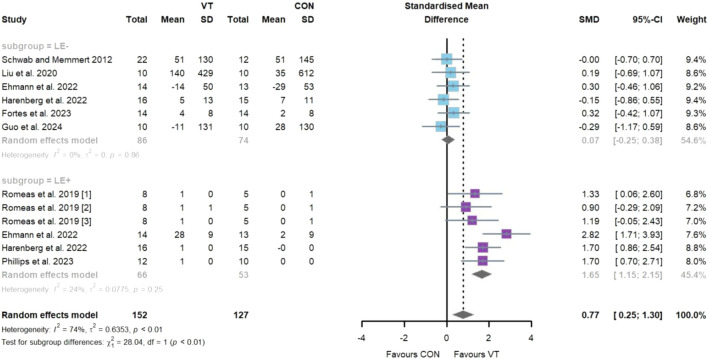
Random-effects meta-analysis of the comparative effects of visual training on visual attention between the “LE−” and “LE+” groups.

Regarding the impact of VT on visual attention (Figure 2), eight studies comprising twelve intervention groups and a total of 269 participants were included. The meta-analysis revealed a statistically significant improvement [SMD = 0.77; 95% CI = (0.25–1.30); *I* ^2^ = 74%; *p* = 0.00], indicating a medium effect size. High heterogeneity was observed. Egger’s test indicated potential publication bias in the primary pooled effect size (*p* = 0.03), supported by asymmetry in the funnel plot (Figure 7a). Sensitivity analysis confirmed the robustness of the pooled estimate (Supplementary Appendix Figure 4.1). Subgroup analysis showed that the “LE−” group did not exhibit a statistically significant improvement [*k* = 6, *n* = 160, SMD = 0.07; 95% CI = (−0.25 to 0.38); *I* ^2^ = 0% (low); *p* > 0.05], while the “LE+” group demonstrated a significant improvement with a large effect size [*k* = 6, *n* = 109, SMD = 1.65; 95% CI = (1.15–2.15); *I* ^2^ = 24% (low); *p* < 0.05]. A significant between-group difference was detected (*p* = 0.00).

**FIGURE 3:**
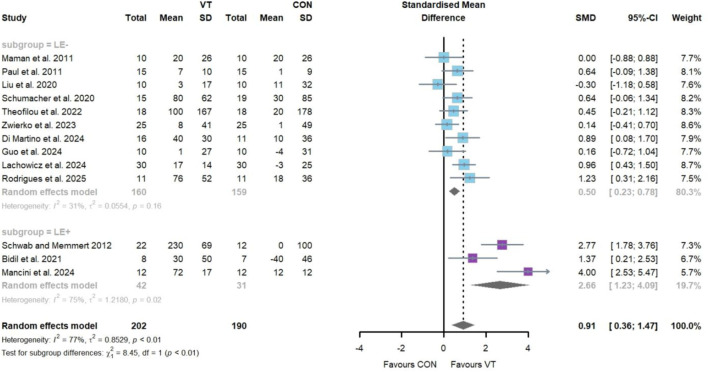
Random-effects meta-analysis of the comparative effects of visual training on reaction time between the “LE−” and “LE+” groups.

Regarding the impact of VT on reaction time (Figure 3), thirteen studies involving thirteen intervention groups and a total of 392 participants were included. The meta-analysis revealed a statistically significant improvement [SMD = 0.91; 95% CI = (0.36–1.47); *I* ^2^ = 77%; *p* = 0.00], indicating a large effect size. Considerable heterogeneity was observed. Egger’s test did not indicate significant publication bias (*p* = 0.07), although visual inspection revealed asymmetry in the funnel plot (Figure 7b). Sensitivity analysis confirmed the robustness of the pooled effect size (Supplementary Appendix Figure 4.2). Subgroup analysis showed that the “LE−” group exhibited a statistically significant improvement with a moderate effect size [*k* = 10, *n* = 319, SMD = 0.50; 95% CI = (0.23–0.78); *I* ^2^ = 31% (moderate); *p* > 0.05], while the “LE+” group demonstrated a large and significant effect [*k* = 3, *n* = 73, SMD = 2.66; 95% CI = (1.23–4.09); *I* ^2^ = 75% (high); *p* > 0.05]. A statistically significant difference was observed between the two subgroups (*p* = 0.00).

**FIGURE 4:**
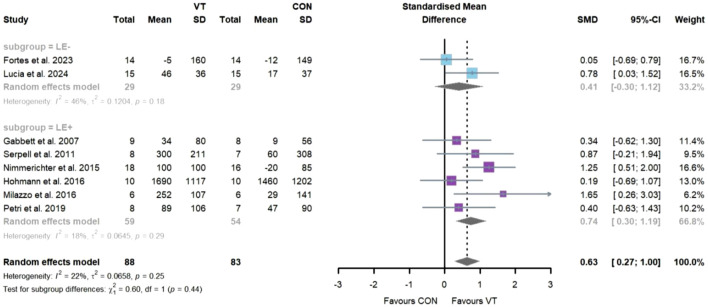
Random-effects meta-analysis of the comparative effects of visual training on decision-making time between the “LE−” and “LE+” groups.

Regarding the impact of VT on decision-making time (Figure 4), eight studies comprising eight intervention groups and a total of 171 participants were included. The meta-analysis showed a statistically significant improvement [SMD = 0.63; 95% CI = (0.27–1.00); *I* ^2^ = 22%; *p* = 0.00], with low heterogeneity observed. Egger’s test indicated no significant publication bias (*p* = 0.55), which was consistent with the symmetrical distribution observed in the funnel plot (Figure 7c). Sensitivity analysis supported the robustness of the pooled effect size (Supplementary Appendix Figure 4.3). Subgroup analysis revealed that the “LE−” group did not show a statistically significant improvement [*k* = 2, *n* = 58, SMD = 0.41; 95% CI = (−0.30–1.12); *I* ^2^ = 46% (moderate); *p* > 0.05], while the “LE+” group exhibited a significant improvement with a moderate effect size [*k* = 6, *n* = 113, SMD = 0.74; 95% CI = (0.30–1.19); *I* ^2^ = 18% (low); *p* < 0.05]. No significant difference was detected between the two subgroups (*p* = 0.44).

**FIGURE 5:**
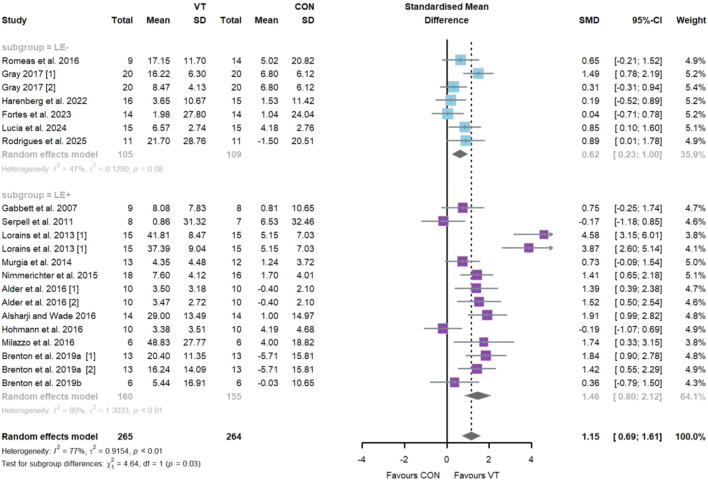
Random-effects meta-analysis of the comparative effects of visual training on decision-making accuracy between the “LE−” and “LE+” groups.

Regarding the impact of VT on decision-making accuracy (Figure 5), seventeen studies comprising twenty-one intervention groups and a total of 471 participants were included. The meta-analysis revealed a statistically significant improvement [SMD = 1.15; 95% CI = (0.69–1.61); *I* ^2^ = 77%; *p* = 0.00], indicating a large effect size. Considerable heterogeneity was observed. Egger’s test indicated potential publication bias (*p* = 0.01), consistent with the asymmetry observed in the funnel plot (Figure 7d). Sensitivity analysis confirmed the robustness of the pooled effect size (Supplementary Appendix Figure 4.4). Subgroup analysis showed that the “LE−” group demonstrated a statistically significant improvement with a moderate effect size [*k* = 7, *n* = 194, SMD = 0.62; 95% CI = (0.23–1.00); *I* ^2^ = 47% (moderate); *p* < 0.05], while the “LE+” group exhibited a significant improvement with a large effect size [*k* = 14, *n* = 277, SMD = 1.46; 95% CI = (0.80–2.12); *I* ^2^ = 80% (considerable); *p* < 0.05]. A statistically significant difference was observed between the two subgroups (*p* = 0.03).

**FIGURE 6:**
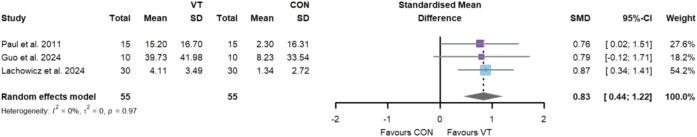
Random-effects meta-analysis of the comparative effects of visual training on eye-hand coordination.

**FIGURE 7:**
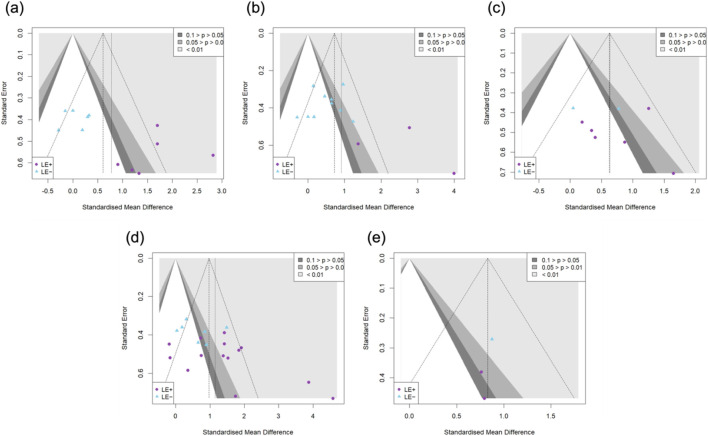
Funnel plots for studies reporting **(a)** visual attention; **(b)** reaction time; **(c)** decision-making time; **(d)** decision-making accuracy; **(e)** eye-hand coordination.

Regarding the impact of VT on eye-hand coordination (Figure 6), three studies comprising three intervention groups and a total of 110 participants were included. The meta-analysis revealed a statistically significant improvement [SMD = 0.83; 95% CI = (0.44–1.22); *I* ^2^ = 0%; *p* = 0.00], indicating a large effect size with low heterogeneity. Egger’s test indicated no evidence of publication bias (*p* = 0.39), which was consistent with the symmetrical distribution observed in the funnel plot (Figure 7e). Sensitivity analysis supported the robustness of the pooled effect size (Supplementary Appendix Figure 4.5). As the number of included studies was fewer than five, subgroup analysis was not conducted.

## 4 Discussion

### 4.1 Influence of “learning effect” on the effectiveness of visual training

This study conducted a systematic subgroup analysis to examine the moderating role of the “learning effect” on the efficacy of VT across various visual-cognitive outcomes. Among the 33 RCTs included, 22 studies met the predefined criteria for the presence of a “learning effect” (LE+). Across four key outcomes—visual attention, reaction time, decision-making time, and decision-making accuracy—the “LE+” group consistently exhibited larger effect sizes compared to the “LE−” group. Notably, significant between-group differences were observed for visual attention (*p* = 0.00), reaction time (*p* = 0.00), and decision-making accuracy (*p* = 0.03).

These findings suggest that when test tools and procedures are structurally similar to the training tasks, participants tend to exhibit better performance, likely due to familiarity with the device interface, stimulus presentation, and response format. Such improvements do not necessarily reflect genuine enhancement of neural processing capabilities but may instead result from task-dependent procedural memory activation or strategic response optimization, known as the “learning effect” (Basner et al., 2020). This also supports the argument of Fransen (Fransen, 2024), who emphasized that the benefits of VT fail to demonstrate robust “far transfer” effects to actual athletic performance. In VT interventions—particularly in perceptual-cognitive and visuomotor coordination training—the “learning effect” has emerged as a systematically overlooked source of bias. It systematically inflates training outcomes through task structural overlap, thereby obscuring the actual extent of neuroplasticity in the visual system (Krasich et al., 2016; Yoon et al., 2019). For example, in the case of visual attention, studies (Romeas et al., 2019; Ehmann et al., 2022; Harenberg et al., 2022; Phillips et al., 2023) in the “LE+” group included multiple object tracking tasks during training and reported significant improvements (SMD = 1.65). In contrast, when training tasks lacked similarity to the tests—even when they contained attention-related components (Ehmann et al., 2022; Harenberg et al., 2022; Guo et al., 2024) —no meaningful improvement was observed (SMD = 0.07). While our previous studies (Guo et al., 2024) reported no significant effects of VT on reaction time, the current analysis showed different results, potentially due to the inclusion of Tier 2 trained individuals who have greater room for improvement compared to athletes. A similar pattern was observed for reaction time: the “LE+” group showed markedly stronger effects, and all three studies (Schwab and Memmert, 2012; Bidil et al., 2021; Mancini et al., 2024) in this group used choice reaction time tasks. Choice reaction time tasks typically involve discriminating and matching multiple stimuli and making rule-based judgments, making them more complex than simple reaction time tasks (Rosenbaum, 2010). As a result, participants may develop specific strategies or response patterns through repeated exposure, relying on strategic responses rather than genuine improvements in neural conduction speed or visual-cognitive processing. In addition, this type of test may be influenced by participants’ compensatory mechanisms for slower responses (Guo et al., 2024), further contributing to inflated test scores.

In decision-making assessments, both response time and accuracy appeared to be affected by the “learning effect”, suggesting that improvements in decision-making following VT may be substantially influenced by test design and task structure. Decision-making inherently involves the rapid identification of external cues, judgment based on experiential rules, and the selection of appropriate behavioral responses—a process requiring the coordination of visual perception (Zhu et al., 2024; Klatt and Smeeton, 2022), working memory (Glavaš et al., 2023; Wu et al., 2025), attentional allocation (Silva et al., 2022), and cognitive control (Heilmann et al., 2024). Decision-making tests often simulate realistic competitive scenarios—such as anticipating an opponent’s movement direction (Milazzo et al., 2016; Petri et al., 2019; Di et al., 2024), predicting ball trajectories (Lucia et al., 2024), or selecting optimal responses from multiple alternatives (Lucia et al., 2024; Mancini et al., 2024; Rodrigues et al., 2025). The complexity of such tasks means that test validity largely depends on the logic and realism of the testing context. However, when test tasks closely resemble the training conditions in terms of stimulus presentation, number and structure of decision options, or feedback mechanisms, participants may develop fixed decision pathways or strategy templates through repeated exposure. Such gains, rooted in familiarity and procedural memory, differ from true cognitive transfer and instead reflect automation in processing specific tasks, rather than improvements in generalized decision-making under dynamic conditions (Cretton et al., 2025). For example, in the “LE+” group, most studies (Gabbett et al., 2007; Lorains et al., 2013; Nimmerichter et al., 2015; Alder et al., 2016; Alsharji and Wade, 2016; Milazzo et al., 2016; Brenton et al., 2019a; Brenton et al., 2019b) employed test stimuli and discrimination formats nearly identical to those used in training—often utilizing the same visual simulation software or platforms. While this setup ensured procedural alignment between training and testing, it also substantially increased the likelihood of test-dependent learning, thereby inflating the observed effect sizes. This bias was particularly evident in decision accuracy, where the “LE+” group exhibited a notably higher effect size compared to the “LE−” group (SMD = 1.46 vs. 0.62), with the between-group difference reaching statistical significance (*p* = 0.03).

Therefore, task structure–dependent performance gains not only compromise the external validity of VT evaluations but also pose challenges for the development of subsequent intervention strategies. If researchers overlook the influence of the “learning effect” on assessment outcomes, they may mistakenly interpret structurally closed and task-specific training protocols as having generalizable transfer value and extend them to other sports or populations. In reality, the true effectiveness of VT hinges on its ability to promote the generalization of cognitive processing and the enhancement of strategic decision-making skills.

### 4.2 Recommendations for research design and outcome assessment

The findings of this study suggest that when test tasks closely resemble training content in structural design, the “learning effect” may substantially inflate the observed benefits of VT, thereby compromising the validity and interpretability of experimental outcomes. This issue is particularly salient in current studies that extensively use digital tools and standardized test methods, where performance gains driven by task familiarity and procedural memory—rather than actual ability—have emerged as a critical source of bias in evaluating training effectiveness (Fransen, 2024). Therefore, proactively identifying and mitigating the influence of “learning effect” during the research design phase has become a crucial prerequisite for improving the methodological quality of VT intervention studies. To this end, researchers should make more deliberate and systematic decisions regarding the structural design of intervention and testing tasks, the selection of outcome measures, and the implementation of evaluation procedures.

First, researchers should avoid selecting test tools and designing tasks that closely resemble the training conditions in terms of interface layout, stimulus type, response format, or feedback mechanisms. When training and testing share the same platform, procedures, or task logic, participants may rely on previously formed procedural strategies during testing, potentially masking the true effects of the intervention on visual–cognitive skills (Lloyd et al., 2025). In contrast, using structurally dissimilar but functionally equivalent heterogenous tasks as assessment tools can better capture transferable improvements and enhance the interpretability and generalizability of research findings. The “learning effect” is typically most pronounced in the early stages of repeated testing and tends to diminish as participants become more familiar with the task (Hammers et al., 2024). Therefore, researchers should carefully plan the timing of interventions and test sessions, ensuring that key evaluations are conducted after participants have adapted to the task and the “learning effect” has stabilized or dissipated. To more accurately assess the true effects of VT, researchers should also prioritize the use of gold-standard visual assessment tools with high reliability and validity. These gold-standard tests should not only demonstrate robust psychometric properties but also distinguish between ability-based improvements and strategy-based gains driven by task familiarity. Additionally, only seven of the included studies conducted retention tests ranging from 1 to 10 weeks post-intervention. To comprehensively evaluate the long-term value of VT, future studies should incorporate retention assessments, which help distinguish short-term strategy-based gains from true neural adaptation and ability consolidation, thereby reflecting the durability and stability of training effects (Willey and Liu, 2018). In addition, future trials should adopt standardized protocols to minimize potential learning effects, such as randomizing device configurations and employing alternate stimulus sets across training and testing.”

The ultimate goal of VT is to enhance sport-specific performance. Therefore, relying solely on laboratory-based visual metrics may be insufficient to fully capture the practical benefits of such training (Fransen, 2024). Study designs should incorporate field-based assessments of sport-specific skills, such as motor responses, decision-making execution, and technical performance under competitive conditions, to evaluate whether improvements in visual abilities effectively transfer to athletic performance. Integrating laboratory-based evaluations with field tests that offer higher ecological validity allows for a more comprehensive assessment of VT outcomes and provides a stronger foundation for optimizing and scaling intervention programs (Laby and Appelbaum, 2021).

### 4.3 Limitations of the present study

The present study has several limitations as follows: (1) Although relatively clear criteria were established to classify the presence of the “learning effect”, this process still involved a degree of subjective judgment. Some included studies lacked detailed reporting of training and testing task characteristics, which may have led to misclassification bias. This subjectivity could have influenced the subgroup comparisons and potentially inflated or underestimated the differences observed between LE+ and LE− groups. Future studies should provide more standardized and transparent reporting of task characteristics to allow for more objective classification and replication across reviews; (2) Although all included participants were experienced athletes at Tier 2 or above, considerable heterogeneity existed in terms of sport type, training background, age, and gender. These factors may influence participants’ receptiveness to training and learning rates, thereby moderating intervention outcomes. Furthermore, variations in training frequency, and intervention duration across studies posed challenges to the accuracy of effect synthesis; (3) This review included only peer-reviewed RCTs published in English-language databases, excluding studies in other languages, which may have introduced both language and publication bias. This restriction could have led to the exclusion of potentially relevant studies published in other languages or in the gray literature, where null or negative findings are more likely to appear. As a result, the pooled estimates presented in this review may be somewhat inflated. Future reviews should consider incorporating multilingual databases and trial registries to reduce the risk of such bias and provide a more comprehensive evidence base. Finally, the potential for small-study effects should also be acknowledged. Although we performed leave-one-out sensitivity analyses to test the robustness of the findings, the limited number of studies and participants in some subgroups increases the likelihood that effect sizes may have been inflated by small-study effects. Therefore, these results should be interpreted with caution until they can be confirmed by larger, well-controlled trials.

## 5 Conclusion

This meta-analysis examined the moderating role of the “learning effect” on the outcomes of VT across different visual–cognitive skills. The results revealed that when the “learning effect” was present, the effectiveness of VT was significantly overestimated. When training and testing tasks shared high structural similarity, participants likely developed task-specific response strategies due to familiarity with the interface, procedures, and task format, leading to inflated test performance that did not reflect genuine improvements in sports vision. These findings suggest that the “learning effect” may constitute a significant source of systematic bias that warrants greater attention and control in future research. To improve the validity and interpretability of future findings, researchers are advised to avoid high structural overlap between training and testing tasks, or to incorporate sufficient familiarization periods and retention tests to distinguish between short-term strategic gains and true neural adaptations.
