# Supplementary material for: Does the “learning effect” caused by digital devices exaggerate sports visual training outcomes? A systematic review and meta-analysis
Source: Front Physiol. 2025 Sep 5;16:1664572. doi: 10.3389/fphys.2025.1664572 (PMC12446229; doi:10.3389/fphys.2025.1664572)
Supplement: Supplementary file 1 [file Supplementaryfile1.docx]

**Supplementary Appendices**

**Title**

Does the “Learning Effect” Caused by Digital Devices Exaggerate Sports Visual Training Outcomes? A Systematic Review and Meta-analysis

**Author List**

Yuqiang Guo^1,2†^, Tinggang Yuan^1*^, Mulin Yang ^3†^, Jinyu Qiu^4^

**Affiliations**

^1^ Science Research Center of Sports Training, China Institute of Sport Science, Beijing, China

^2^ School of Physical Education, Shanghai University of Sport

^3^ Physical Fitness Training Research Center, China Institute of Sport Science

^4^ Xinxin School, The Affiliated High School of Peking University

**Context**

[RISMA 2020 Checklist 1](#_Toc207711546)

[Appendix 1 – Search Strategy 6](#_Toc207711547)

[Appendix 2 – The Characteristics of the Studies Included 7](#_Toc207711548)

[Appendix 3 – Risk of Bias 15](#_Toc207711549)

[Appendix 4 – Sensitivity analysis 16](#_Toc207711550)

[References 18](#_Toc207711551)

# RISMA 2020 Checklist

| **Section and Topic** | **Item #** | **Checklist item** | **Location where item is reported** |
| --- | --- | --- | --- |
| **TITLE** | | |  |
| Title | 1 | Identify the report as a systematic review. | Manuscript Page 01 |
| **ABSTRACT** | | |  |
| Abstract | 2 | See the PRISMA 2020 for Abstracts checklist. | Manuscript Page 01 |
| **INTRODUCTION** | | |  |
| Rationale | 3 | Describe the rationale for the review in the context of existing knowledge. | Manuscript Page 02 |
| Objectives | 4 | Provide an explicit statement of the objective(s) or question(s) the review addresses. | Manuscript Page 02 |
| **METHODS** | | |  |
| Eligibility criteria | 5 | Specify the inclusion and exclusion criteria for the review and how studies were grouped for the syntheses. | Manuscript Page 02-04 |
| Information sources | 6 | Specify all databases, registers, websites, organisations, reference lists and other sources searched or consulted to identify studies. Specify the date when each source was last searched or consulted. | Manuscript Page 02 |
| Search strategy | 7 | Present the full search strategies for all databases, registers and websites, including any filters and limits used. | Supplementary Material Appendix 1 |
| Selection process | 8 | Specify the methods used to decide whether a study met the inclusion criteria of the review, including how many reviewers screened each record and each report retrieved, whether they worked independently, and if applicable, details of automation tools used in the process. | Manuscript Page 02 |
| Data collection process | 9 | Specify the methods used to collect data from reports, including how many reviewers collected data from each report, whether they worked independently, any processes for obtaining or confirming data from study investigators, and if applicable, details of automation tools used in the process. | Manuscript Page 04 |
| –-Data items | 10a | List and define all outcomes for which data were sought. Specify whether all results that were compatible with each outcome domain in each study were sought (e.g. for all measures, time points, analyses), and if not, the methods used to decide which results to collect. | Manuscript Page 04 |
|  | 10b | List and define all other variables for which data were sought (e.g. participant and intervention characteristics, funding sources). Describe any assumptions made about any missing or unclear information. | Manuscript Page 04 |
| Study risk of bias assessment | 11 | Specify the methods used to assess risk of bias in the included studies, including details of the tool(s) used, how many reviewers assessed each study and whether they worked independently, and if applicable, details of automation tools used in the process. | Manuscript Page 04 |
| Effect measures | 12 | Specify for each outcome the effect measure(s) (e.g. risk ratio, mean difference) used in the synthesis or presentation of results. | Manuscript Page 04, 05 |
| Synthesis methods | 13a | Describe the processes used to decide which studies were eligible for each synthesis (e.g. tabulating the study intervention characteristics and comparing against the planned groups for each synthesis (item #5)). | Supplementary Material Appendix 2 |
|  | 13b | Describe any methods required to prepare the data for presentation or synthesis, such as handling of missing summary statistics, or data conversions. | Manuscript Page 04 |
|  | 13c | Describe any methods used to tabulate or visually display results of individual studies and syntheses. | Manuscript Page 05 |
|  | 13d | Describe any methods used to synthesize results and provide a rationale for the choice(s). If meta-analysis was performed, describe the model(s), method(s) to identify the presence and extent of statistical heterogeneity, and software package(s) used. | Manuscript Page 04, 05 |
|  | 13e | Describe any methods used to explore possible causes of heterogeneity among study results (e.g. subgroup analysis, meta-regression). | Manuscript Page 05 |
|  | 13f | Describe any sensitivity analyses conducted to assess robustness of the synthesized results. | Manuscript Page 05 |
| Reporting bias assessment | 14 | Describe any methods used to assess risk of bias due to missing results in a synthesis (arising from reporting biases). | Manuscript Page 04 |
| Certainty assessment | 15 | Describe any methods used to assess certainty (or confidence) in the body of evidence for an outcome. | / |
| **RESULTS** | | |  |
| Study selection | 16a | Describe the results of the search and selection process, from the number of records identified in the search to the number of studies included in the review, ideally using a flow diagram. | Manuscript Page 05 |
|  | 16b | Cite studies that might appear to meet the inclusion criteria, but which were excluded, and explain why they were excluded. | / |
| Study characteristics | 17 | Cite each included study and present its characteristics. | Supplementary Material Appendix 2 |
| Risk of bias in studies | 18 | Present assessments of risk of bias for each included study. | Supplementary Material Appendix 3 |
| Results of individual studies | 19 | For all outcomes, present, for each study: (a) summary statistics for each group (where appropriate) and (b) an effect estimate and its precision (e.g. confidence/credible interval), ideally using structured tables or plots. | Manuscript Figure 2-6 |
| Results of syntheses | 20a | For each synthesis, briefly summarise the characteristics and risk of bias among contributing studies. | Manuscript Page 05, 06 |
|  | 20b | Present results of all statistical syntheses conducted. If meta-analysis was done, present for each the summary estimate and its precision (e.g. confidence/credible interval) and measures of statistical heterogeneity. If comparing groups, describe the direction of the effect. | Manuscript Page 06-09 |
|  | 20c | Present results of all investigations of possible causes of heterogeneity among study results. | Manuscript Page 06-09 |
|  | 20d | Present results of all sensitivity analyses conducted to assess the robustness of the synthesized results. | Manuscript Page 06-09  Supplementary Material Appendix 4 |
| Reporting biases | 21 | Present assessments of risk of bias due to missing results (arising from reporting biases) for each synthesis assessed. | Manuscript Page 06-09 |
| Certainty of evidence | 22 | Present assessments of certainty (or confidence) in the body of evidence for each outcome assessed. | / |
| **DISCUSSION** | | |  |
| Discussion | 23a | Provide a general interpretation of the results in the context of other evidence. | Manuscript Page 09, 10 |
|  | 23b | Discuss any limitations of the evidence included in the review. | Manuscript Page 11 |
|  | 23c | Discuss any limitations of the review processes used. | Manuscript Page 11 |
|  | 23d | Discuss implications of the results for practice, policy, and future research. | Manuscript Page 10, 11 |
| **OTHER INFORMATION** | | |  |
| Registration and protocol | 24a | Provide registration information for the review, including register name and registration number, or state that the review was not registered. | Manuscript Page 02 |
|  | 24b | Indicate where the review protocol can be accessed, or state that a protocol was not prepared. | Manuscript Page 02 |
|  | 24c | Describe and explain any amendments to information provided at registration or in the protocol. | / |
| Support | 25 | Describe sources of financial or non-financial support for the review, and the role of the funders or sponsors in the review. | Manuscript Page 12 |
| Competing interests | 26 | Declare any competing interests of review authors. | Manuscript Page 12 |
| Availability of data, code and other materials | 27 | Report which of the following are publicly available and where they can be found: template data collection forms; data extracted from included studies; data used for all analyses; analytic code; any other materials used in the review. | Manuscript Page 12 |

# Appendix 1 – Search Strategy

***Web of Science (Core Collection) (n = 536)***

TS=(“visual training” OR “vision training” OR “eye training” OR “visuomotor training” OR “visual motor training” OR “perceptual training” OR “perceptual-cognitive training” OR “temporal occlusion training” OR “strobe training” OR “stroboscopic training” OR “virtual reality training” OR “VR training” OR “visual-spatial training” OR “visual search training” OR “multiple object tracking training”) AND TS=(“randomized controlled trial” OR “random allocation” OR “RCT” OR “randomized” OR “randomly”)

***PubMed (n = 598)***

(“visual training”[Title/Abstract] OR “vision training”[Title/Abstract] OR “eye training”[Title/Abstract] OR “visuomotor training”[Title/Abstract] OR “visual motor training”[Title/Abstract] OR “perceptual training”[Title/Abstract] OR “perceptual-cognitive training”[Title/Abstract] OR “temporal occlusion training”[Title/Abstract] OR “strobe training”[Title/Abstract] OR “stroboscopic training”[Title/Abstract] OR “virtual reality training”[Title/Abstract] OR “VR training”[Title/Abstract] OR “visual-spatial training”[Title/Abstract] OR “visual search training”[Title/Abstract] OR “multiple object tracking training”[Title/Abstract]) AND (“randomized controlled trial”[Publication Type] OR “random allocation”[MeSH Terms] OR “RCT”[Title/Abstract] OR “randomized”[Title/Abstract] OR “randomly” [Title/Abstract])

***MEDLINE (n = 1150) and SPORTDiscus (n = 705) through EBSCOhost***

TX ((“visual training” OR “vision training” OR “eye training” OR “visuomotor training” OR “visual motor training” OR “perceptual training” OR “perceptual-cognitive training” OR “temporal occlusion training” OR “strobe training” OR “stroboscopic training” OR “virtual reality training” OR “VR training” OR “visual-spatial training” OR “visual search training” OR “multiple object tracking training”)) AND TX ((“randomized controlled trial” OR “random allocation” OR “RCT” OR “randomized” OR “randomly”))

***Cochrane Library (n = 789)***

#1 “visual training” OR “vision training” OR “eye training” OR “visuomotor training” OR “visual motor training” OR “perceptual training” OR “perceptual-cognitive training” OR “temporal occlusion training” OR “strobe training” OR “stroboscopic training” OR “virtual reality training” OR “VR training” OR “visual-spatial training” OR “visual search training” OR “multiple object tracking training” 1135

#2 “randomized controlled trial” OR “random allocation” OR “RCT” OR “randomized” OR “randomly” 1287931

#3 #1 AND #2 789

# Appendix 2 – The Characteristics of the Studies Included

Table 2.1 Summary of characteristics of all studies meeting the inclusion criteria

| **Study** | **Population** | **Group (n)** | **Training type** | **Intervention characteristics** | **Measures** | **Is there a “learning effect” caused by digital devices?** | **Is there a “retention test”?** |
| --- | --- | --- | --- | --- | --- | --- | --- |
| Gabbett et al. (2007) | Female  Age = 19 ± 6  Softball  Subelite | EXP = 9  PLA = 8  CON = 8 | EXP: PCT  PLA: Left/Right arrows  CON: No additional training | 3 times/ week  4 weeks  10 min | DRA^#^  DRT^#^ | ① Training and testing used highly similar digital devices (video-based presentations).  ② Both tasks employed temporally occluded batter-perspective videos with identical occlusion timing and decision targets. | 4 weeks |
| Maman et al. (2011) | Male  Age = 18 - 25  Tennis  Trained | EXP = 10  PLA = 10  CON = 10 | EXP: VCT  PLA: Reading and watching  CON: No additional training | 3 times/ week  8 weeks  30 min | RT | None | None |
| Paul et al. (2011) | Mixed  Age = 18 - 28  Table tennis  Trained | EXP = 15  PLA = 15  CON = 15 | EXP: VCT  PLA: Reading and watching  CON: No additional training | 3 times/ week  8 weeks  30 min | RT  EHC^#^ | ① Training and testing used highly similar digital devices (Vienna Testing System, Schuhfried, Austria) for eye-hand coordination tasks.  ② Both tasks employed identical on-screen ball-tracing exercises with consistent boundary avoidance objectives and dual-joystick control mechanisms. | None |
| Serpell et al. (2011) | Age = under 20  Rubgy  Subelite | EXP = 8  CON = 7 | EXP: PCT  CON: No additional training | 2 times/ week  3 weeks  15 min | DRA^#^  DRT^#^ | ① Training and testing used highly similar digital devices (Sanyo PLC-XU48 projector, 2×2 m screen, and ASUS laptop) for reactive agility tasks.  ② Both tasks employed identical video-based occlusion paradigms with consistent kinematic cue recognition objectives and direction-change response mechanisms. | None |
| Schwab and Memmert (2012) | Male  Age =14 ± 2  Field hockey  Trained | EXP = 22  CON = 12 | EXP: VCT  CON: No additional training | 3 times/ week  6 weeks  45 min | RT^#^  VA | ① Training and testing of Reaction Time were conducted on the same digital device (Dynavision D2®);  ② Both training and testing using similar task formats. | 6 weeks |
| Lorains et al. (2013) | Age =22 ± 3  Soccer  Elite | EXP1 = 15  EXP2 = 15  CON = 15 | EXP1: Fast speed PCT  EXP2: Normal speed PCT  CON: No additional training | 1 times/ week  5 weeks  20 - 30 min | DRA | ① Training and testing used identical software on laptop screens.  ② Both training and testing using similar task formats. | 2 week  10 week |
| Murgia et al. (2014) | Male  Age = 16 ± 2  Soccer  Elite | EXP = 13  PLA = 13  CON = 12 | EXP: PCT  PLA: Watching DVD  CON: No additional training | Total 11 - 6 times  6 weeks  Total 212 - 175 minutes | DRA^#^ | ① Training (DVD on participants’ computers) and testing (ASUS X52J laptop) used highly similar digital devices (computers).  ② Training and testing tasks both used temporally occluded goalkeeper-perspective penalty videos with same occlusion timing and prediction targets. | None |
| Nimmerichter et al. (2015) | Male  Age = 14 ± 0  Soccer  Subelite | EXP = 18  CON = 16 | EXP: PCT  CON: No additional training | 3 times/ week  6 weeks  6 min | DRT^#^  DRA^#^ | ① Training (Lenovo ThinkPad T420s laptops with VLC Media Player) and testing (computer with Utilius® fairplay 5 software) used highly similar digital devices (computers).  ② Training and testing tasks both used first-person perspective one-on-one situation videos with identical occlusion timing (at defensive tackle initiation) and prediction targets (left/right direction). | None |
| Alder et al. (2016) | Mixed  Age = 21 ± 2  Badminton  Elite | EXP1 = 10  EXP2 = 10  CON = 10 | EXP1: High-anxiety PCT  EXP2: Low-anxiety PCT  CON: No additional training | 1 times/ week  3 weeks  30 min | DRA^#^ | ① Training and testing used highly similar devices (2.74×3.66 m screens, Adobe Premiere) for video occlusion tasks.  ② Both tasks used first-person badminton serve videos with identical shuttle-racket contact occlusion. | None |
| Alsharji and Wade (2016) | Male  Subelite: Age = 17 ± 1  Elite: Age = 29 ± 5  Handball  Subelite and elite | EXP = 14  PLA = 14  CON = 14 | EXP: PCT  PLA: Watching  CON: No additional training | 7 times/ week  1 weeks  20 min | DRA^#^ | ① Training and testing used highly similar devices (iPads and large screens) for video presentation.  ② Both tasks used goalkeeper-view 7-m throw videos with same occlusion timing and direction targets. | None |
| Hohmann et al. (2016) | Male  Age = 15 ± 1  Handball  Elite | EXP1 = 10  EXP2 = 10  CON = 10 | EXP1: PCT  EXP2: Tactic board training  CON: No additional training | 1 times/ week  6 weeks  30 min | DRA^#^  DRT^#^ | ① Training and testing used similar devices (3D projection/2D screens) for video tasks.  ② Both used first-person handball videos with same occlusion points and decision targets. | 4 weeks |
| Milazzo et al. (2016) | Female  Age =16 ± 1  Karate  Subelite | EXP = 6  PLA = 6  CON = 6 | EXP: PCT  PLA: Motor training  CON: No additional training | 4 times/ week  3 weeks  8 min | DRA^#^  DRT^#^ | ① Training and testing used similar devices (Sony projector and projection wall) for video tasks.  ② Both used first-person karate fight videos with same occlusion points (at attack initiation) and decision targets (attack direction). | None |
| Romeas et al. (2016) | Male  Age = 22 ± 1  Soccer  Subelite | EXP = 9  CON = 14 | EXP: MOT  Active CON: video watching  Passive CON: No additional training | 2 times/ week  5 weeks  40 min | DRA | None | None |
| Gray (2017) | Male  Age = 17 – 18  Baseball  Trained | EXP1 = 20  EXP3 = 20  EXP2 = 20  CON = 20 | EXP1: VRT  EXP2: VRT  EXP3: Real batting practice  CON: No additional training | 2 times/ week  6 weeks  45 min | DRA | None | 1 month |
| Brenton et al. (2019) | Male  Age = 18 - 36  Criket  Trained | EXP1 = 13  EXP2 = 13  CON = 13 | EXP1: PCT  EXP2: VCT  CON: No additional training | 2 times/ week  5 weeks  EXP1: 10 min  EXP2: 20 min | DRA^#^ | ① Training used a Dell laptop and Epson projector to present point-light displays; testing employed the same projection system with a 1.4 m×1.4 m screen, both visual-motor interactive devices.  ② Both training and testing) required ball type prediction from temporally occluded videos, using identical stimulus materials and response modes. | None |
| Brenton et al. (2019) | Male  EXP: Age = 24 ± 3  CON: Age = 22 ± 3  Criket  Trained | EXP = 8  CON = 7 | EXP: VCT  CON: No additional training | 2 times/ week  4 weeks  15 min | DRA^#^ | ① Training used a Dell laptop and Epson projector to present point-light displays; testing employed the same projection system with a 1.4 m×1.4 m screen, both visual-motor interactive devices.  ② Both training and testing required ball type prediction from temporally occluded videos, using identical stimulus materials and response modes. | None |
| Petri et al. (2019) | Mixed  Age = 13-18  Karate  Elite | EXP = 8  CON = 7 | EXP: VRT  CON: No additional training | 2 times/ week  5 weeks  10-15 min | DRT^#^ | ① Training used Oculus Rift DK2 HMD and hand trackers; testing applied high-speed cameras and projector, both electronic visual devices with similar functions.  ② Both training and testing tasks required reactions to virtual opponent attacks, using identical attack types and response patterns for close task structure matching. | None |
| Romeas et al. (2019) | Mixed  Age = 23 ± 3  Badminton  Subelite | EXP1 = 8  EXP2 = 8  EXP3 = 8  CON = 5 | EXP1: MOT  EXP2: MOT + Decision-making task  EXP3: MOT + Decision-making task  CON: No additional training | Total 9 times  6 weeks  30 min | VA^#^ | ① Training used the EON Icube™ system with projectors and active shutter glasses; testing employed the same immersive virtual environment, both visual-motor interactive devices.  ② Both training and testing required 3D-MOT tracking combined with decision-making tasks, using identical stimulus materials (virtual spheres or point-light walkers) and response modes (motor actions or visual judgments). | None |
| Liu et al. (2020) | Male  Age = NR  Baseball  Elite | EXP = 10  CON = 10 | EXP: VCT + SVT  CON: Vision therapy trials | 3 times/ week  10 weeks  30 min | VA  RT | ① Both training and testing adopted the same digital training device Senaptec Sensory Station. | None |
| Schumacher et al. (2020) | Male  Age = 12 – 13  Soccer  Elite | EXP = 15  CON = 19 | EXP: PCT  CON: | 1 times/ week  8 weeks  20 min | RT | None | None |
| Bidil et al. (2021) | Mixed  Age = 21 ± 3  Badminton  Elite | EXP = 8  CON = 7 | EXP: VCT  CON: No additional training | 4 times/ week  8 weeks  180 min | RT^#^ | ① Training and testing used the same FitLight Trainer™ system with consistent parameters.  ② Training tasks replicated badminton-specific reaction tests in structure and mode. | None |
| Ehmann et al. (2022) | Mixed  Age = 12 ± 1  Soccer  Trained | EXP = 14  PLA = 15  CON = 13 | EXP: MOT  PLA: Video watching  CON: No additional training | 2 times/ week  5 weeks  20 min | VA^#^  VA | ① MOT Training and testing used the same Helix-Arena system with consistent parameters.  ② Training tasks replicated 360-MOT test structures and modes in soccer-specific scenarios. | None |
| Harenberg et al. (2022) | Mixed  Age = 19 ± 1  Soccer  Subelite | EXP = 16  CON = 15 | EXP: MOT  CON: 3D video watching | Total 10 times  4 weeks  25 min | VA^#^  VA  DRA | ① Both training and testing used the same Neurotracker 3D MOT software and equipment.  ② Training tasks closely matched testing tasks in structure and mode of 3D object tracking. | None |
| Theofilou et al. (2022) | Male  Age = 10 -15  Soccer  Trained | EXP = 18  CON = 18 | EXP: VCT  CON: No additional training | 5 times/ week  6 months  15 min | RT | None | None |
| Fortes et al. (2023) | Male  Age = 25 ± 5  Soccer  Elite | EXP = 14  CON = 14 | EXP: SVT  CON: Regular training | 3 times/ week  8 weeks  20 min | RT  DRA  VA | None | None |
| Phillips et al. (2023) | Female  Age = 18 - 25  Soccer  Elite | EXP = 12  CON = 10 | EXP: MOT  CON: No additional training | Total 10 times  4 weeks  6 - 8 min | VA^#^ | ① Both training and testing used the same NeuroTracker 3D-MOT equipment.  ② Training tasks matched testing tasks in structure, tracking 4 balls in a 3D cube. | None |
| Zwierko et al. (2023) | Mixed  EXP: Age = 16 ± 1  CON: Age = 17 ± 1  Volleyball  Elite | EXP = 25  CON = 25 | EXP: SVT  CON: Non-stroboscopic training | 3 times/ week  6 weeks  25 - 30 min | RT | None | 4 weeks |
| Di Martino et al. (2024) | Mixed  Age = 17 ± 4  Fencer  Elite | EXP = 16  CON = 11 | EXP: VCT  CON: Regular training | 2 times/ week  6 weeks  12 min | RT | None | None |
| Guo et al. (2024) | Mixed  Age = 25 ± 5  Skeet shooter  Elite | EXP = 10  CON = 10 | EXP: VCT  CON: Target tracking training | 2 times/ week  6 weeks  60 min | VA  RT  EHC^#^ | ① Both training and testing used the same Senaptec Sensory Station equipment.  ② Training tasks closely matched testing tasks in structure and mode. | None |
| Lachowicz et al. (2024) | Mixed  Age = 24 ± 4  Esports  Trained | EXP = 30  CON = 30 | EXP: VRT  CON: No additional training | 8 consecutive weekdays  15 min | RT  EHC | None | 31 days |
| Lucia et al. (2024) | Mixed  Age = 16 ± 1  Basketball  Elite | EXP = 15  CON = 15 | EXP: VCT  CON: Regular training | 2 times/ week  5 weeks  30 min | DRT  DRA | None | None |
| Mancini et al. (2024) | Female  EXP: Age = 20 ± 1  CON: Age = 20 ± 1  Volleyball  Elite | EXP = 12  CON = 12 | EXP: VCT  CON: Regular training | 3 times/ week  6 weeks | RT | ① Both training (ReactionX) and testing (Fitlight) used LED systems for reaction time tasks.  ② Training tasks matched testing tasks in visual stimulus-response structure and mode. | None |
| Rodrigues et al. (2025) | Mixed  Age = 22 ± 2  Soccer  Subelite | EXP = 11  CON = 11 | EXP: VCT  CON: Regular training | 2 times/ week  6 weeks  30 min | RT  DRA | None | None |

Note:

(1) EXP: experimental group; PLA: placebo group; CON: control group.

(2) SVT: stroboscopic visual training; PCT: perceptual-cognitive training; VRT: virtual reality training; VCT: visuomotor coordination training; MOT: multiple object tracking training; VRT: virtual reality training

(3) RT: reaction time; DRA: decision-making response accuracy; DRT:decision-making response time; VA: visual attention; EHC: eye-hand coordination.

(4) ^#^ indicates that this index is interfered by the “learning effect”, affecting the results of its training benefits.

(5) ① The explanation for the study meeting Criterion 1: the digital device used for training and testing was identical or highly similar; ② The explanation for the study meeting Criterion 2: the structure and mode of the training task closely matched those of the outcome measure.

(6) NR: Not reported.

# Appendix 3 – Risk of Bias

Figure 3.1 Risk of Bias 2 assessment for each study


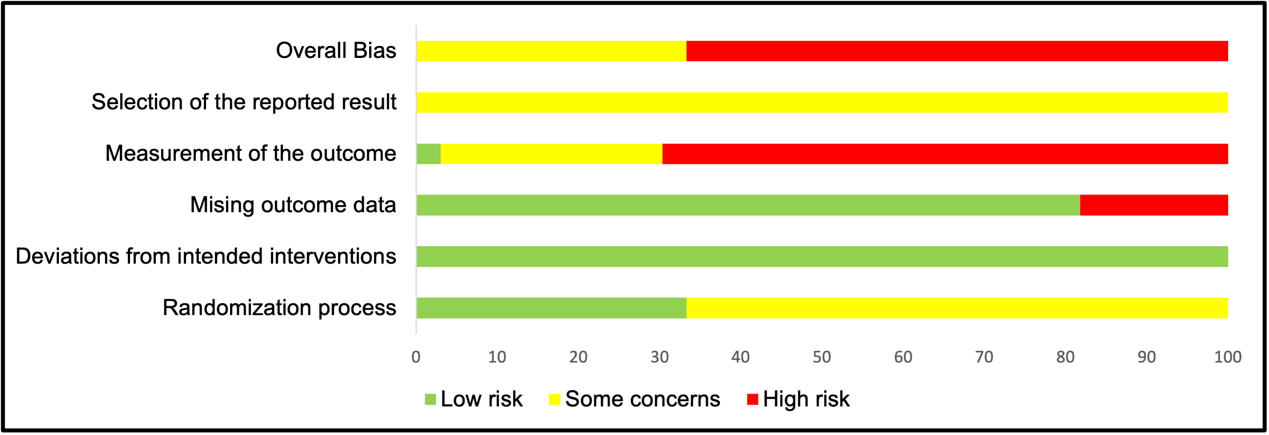


Figure 3.2 Risk of Bias 2 indicated by percentages of assessed biases across all included studies

# Appendix 4 – Sensitivity analysis


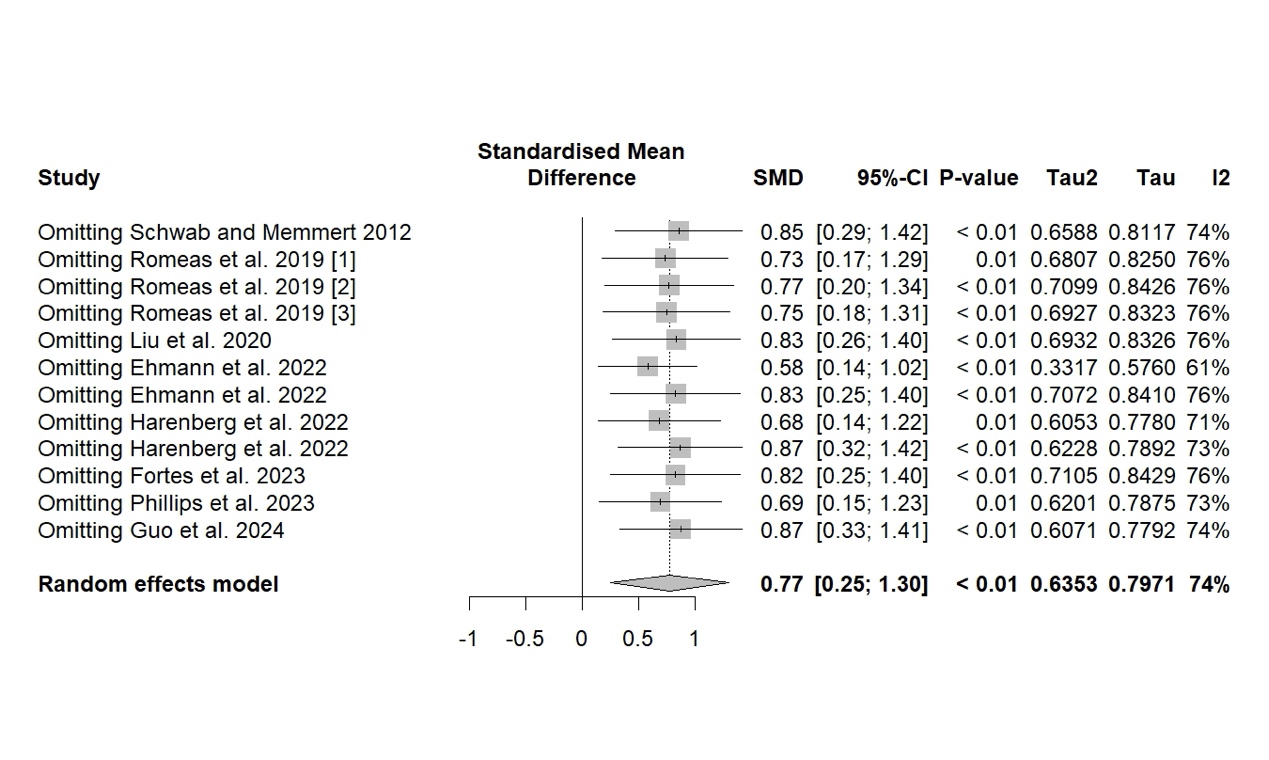


Figure 4.1 Visual Attention


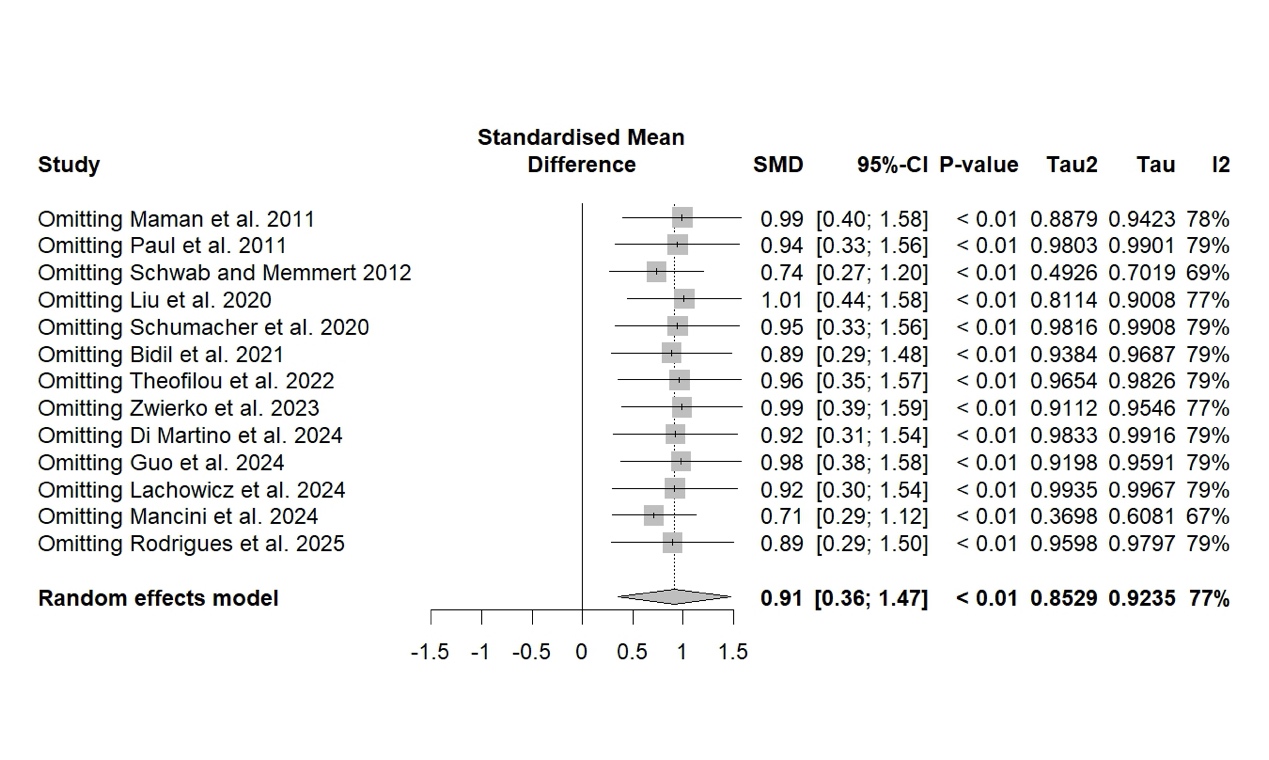


Figure 4.2 Reaction Time


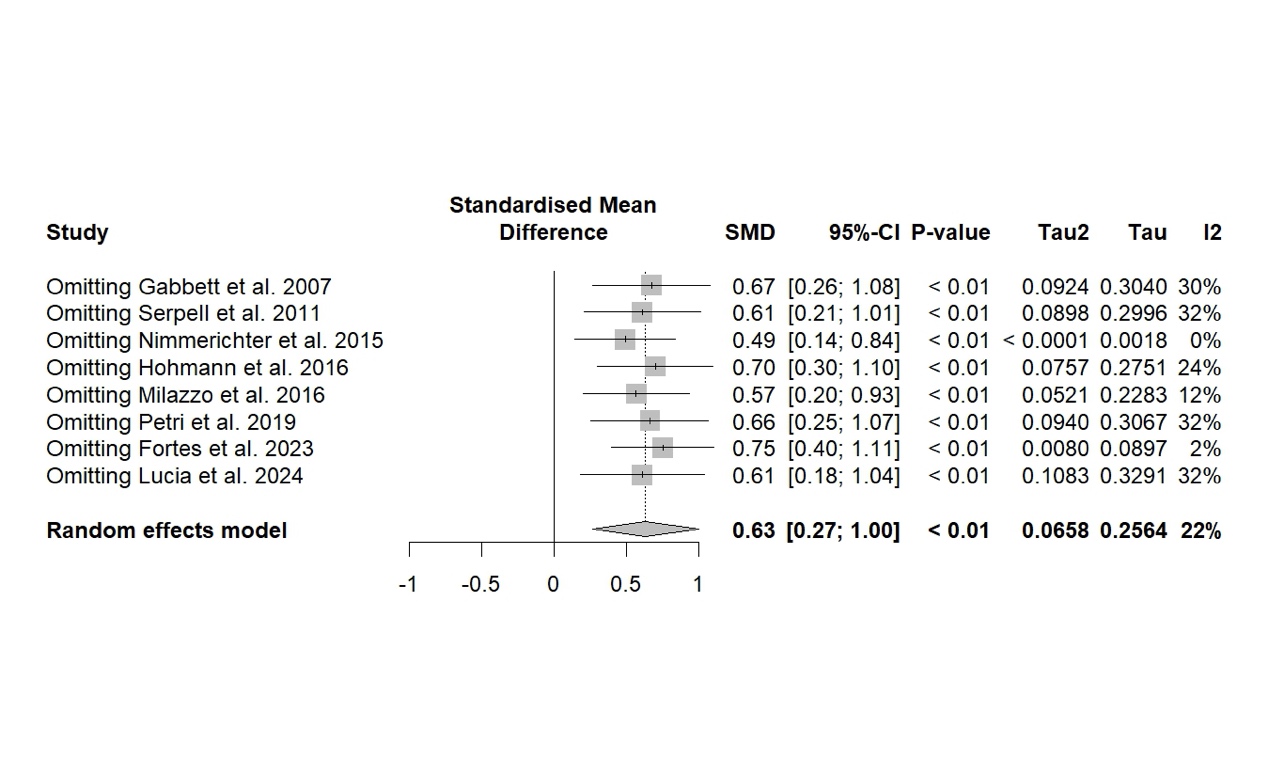


Figure 4.3 Decision-making Time


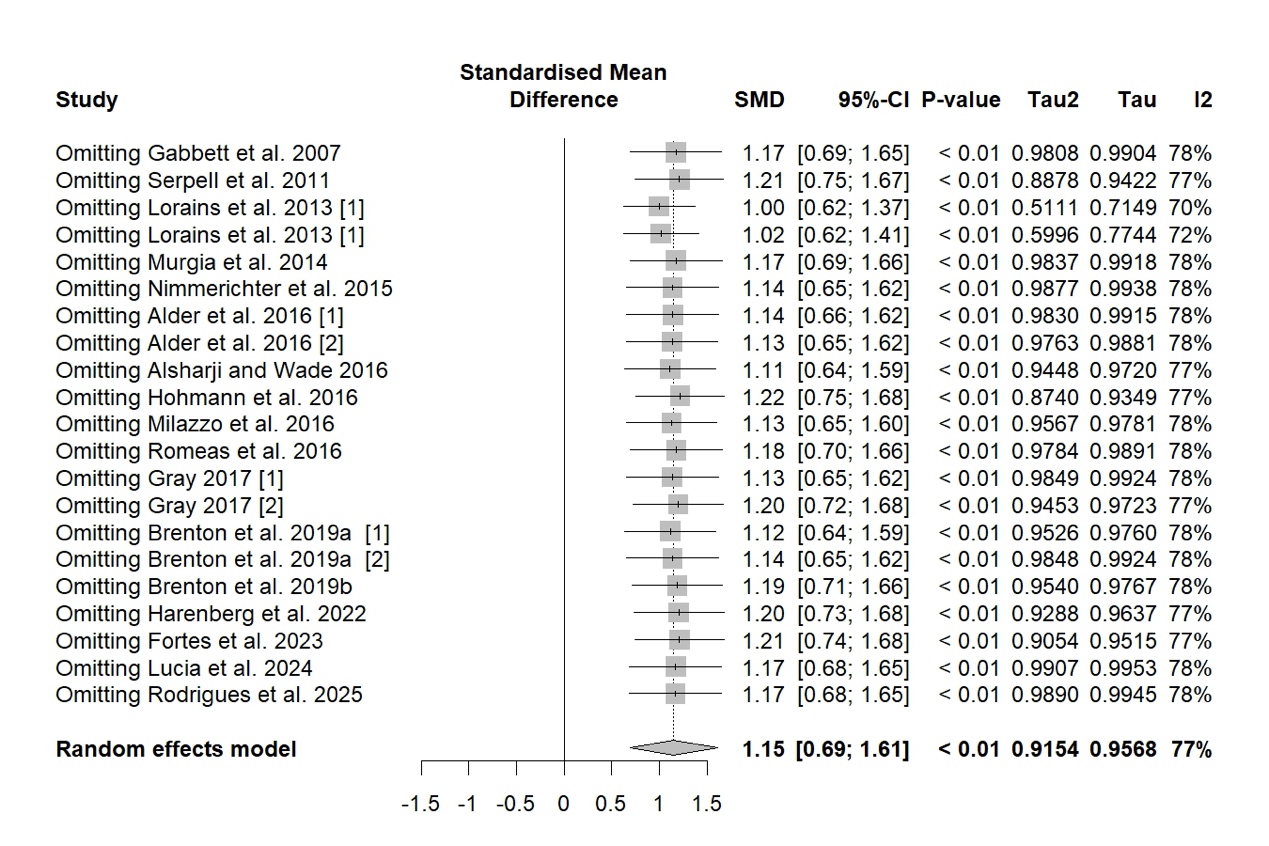


Figure 4.4 Decision-making Accuracy


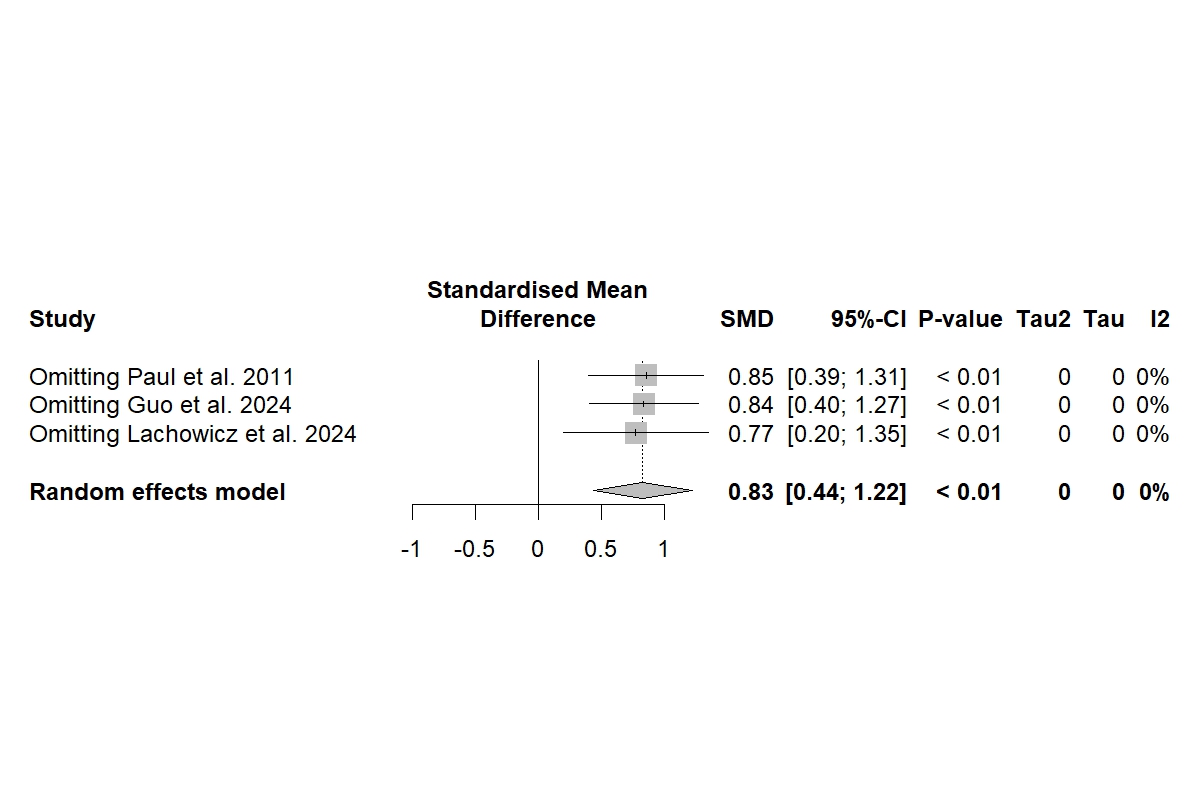


Figure 4.5 Eye-hand Coordination

# References

Alder, D., P. R. Ford, J. Causer and A. M. Williams (2016). "The Effects of High- and Low-Anxiety Training on the Anticipation Judgments of Elite Performers." J Sport Exerc Psychol **38**(1): 93-104.

Alsharji, K. E. and M. G. Wade (2016). "Perceptual training effects on anticipation of direct and deceptive 7-m throws in handball." Journal of sports sciences **34**(2): 155-162.

Bidil, S., B. Arslan, K. Bozkurt, S. C. DÜZova, B. S. ÖRs, E. Onarici GÜNgÖR and D. ŞİMŞEk (2021). "Investigation of the Effect of Badminton-Specific 8 Week Reaction Training on Visual-Motor Reaction Time and Visual Cognitive Dual Task." Turkiye Klinikleri Journal of Sports Sciences **13**(1): 33-40.

Brenton, J., S. Muller and A. Dempsey (2019). "Visual-Perceptual Training With Acquisition of the Observed Motor Pattern Contributes to Greater Improvement of Visual Anticipation." Journal of Experimental Psychology-Applied **25**(3): 333-342.

Brenton, J., S. Müller and A. G. Harbaugh (2019). "Visual-perceptual training with motor practice of the observed movement pattern improves anticipation in emerging expert cricket batsmen." J Sports Sci **37**(18): 2114-2121.

Di Martino, G., S. Giommoni, F. Esposito, D. Alessandro, C. Della Valle, E. Iuliano, G. Fiorilli, G. Calcagno and A. di Cagno (2024). "Enhancing Focus and Short Reaction Time in Épée Fencing: The Power of the Science Vision Training Academy System." J Funct Morphol Kinesiol **9**(4).

Ehmann, P., A. Beavan, J. Spielmann, J. Mayer, L. Ruf, S. Altmann, L. Forcher, N. Klever, S. Rohrmann, C. Nuß and C. Englert (2022). "Perceptual-cognitive performance of youth soccer players in a 360°-environment – An investigation of the relationship with soccer-specific performance and the effects of systematic training." Psychology of Sport &amp; Exercise **61**: N.PAG-N.PAG.

Fortes, L. S., H. Faro, J. Faubert, C. G. Freitas-Júnior, D. d. Lima-Junior and S. S. Almeida (2023). "Repeated stroboscopic vision training improves anticipation skill without changing perceptual-cognitive skills in soccer players." Applied neuropsychology. Adult: 1-15.

Gabbett, T., M. Rubinoff, L. Thorburn and D. Farrow (2007). "Testing and Training Anticipation Skills in Softball Fielders." International Journal of Sports Science &amp; Coaching **2**(1): 15-24.

Gray, R. (2017). "Transfer of Training from Virtual to Real Baseball Batting." Frontiers in psychology **Volume 8 - 2017**.

Guo, Y., T. Yuan, J. Peng, L. Deng and C. Chen (2024). "Impact of sports vision training on visuomotor skills and shooting performance in elite skeet shooters." Frontiers in human neuroscience **18**.

Harenberg, S., Z. McCarver, J. Worley, D. Murr, J. Vosloo, R. S. Kakar, R. McCaffrey, K. Dorsch and O. Hoener (2022). "The effectiveness of 3D multiple object tracking training on decision-making in soccer." Science and Medicine in Football **6**(3): 355-362.

Hohmann, T., H. Obelöer, N. Schlapkohl and M. Raab (2016). "Does training with 3D videos improve decision-making in team invasion sports?" Journal of sports sciences **34**(8): 746-755.

Lachowicz, M., A. Serweta-Pawlik, A. Konopka-Lachowicz, D. Jamro and G. Zurek (2024). "Amplifying Cognitive Functions in Amateur Esports Athletes: The Impact of Short-Term Virtual Reality Training on Reaction Time, Motor Time, and Eye-Hand Coordination." Brain sciences **14**(11).

Liu, S., L. M. Ferris, S. Hilbig, E. Asamoa, J. L. LaRue, D. Lyon, K. Connolly, N. Port and L. G. Appelbaum (2020). "Dynamic vision training transfers positively to batting practice performance among collegiate baseball batters." Psychology of sport and exercise **51**.

Lorains, M., K. Ball and C. MacMahon (2013). "An above real time training intervention for sport decision making." Psychology of sport and exercise **14**(5): 670-674.

Lucia, S., M. Digno, I. Madinabeita and F. Di Russo (2024). "Integration of cognitive-motor dual-task training in physical sessions of highly-skilled basketball players." Journal of sports sciences **42**(18): 1695-1705.

Maman, P., S. Gaurang and J. S. Sandhu (2011). "THE EFFECT OF VISION TRAINING ON PERFORMANCE IN TENNIS PLAYERS." Serbian Journal of Sports Sciences **5**(1): 11-16.

Mancini, N., M. Di Padova, R. Polito, S. Mancini, A. Dipace, A. Basta, D. Colella, P. Limone, G. Messina, M. Monda, A. Monda, M. A. Guerriero, A. Messina and F. Moscatelli (2024). "The Impact of Perception–Action Training Devices on Quickness and Reaction Time in Female Volleyball Players." Journal of Functional Morphology &amp; Kinesiology **9**(3): 147.

Milazzo, N., D. Farrow and J. F. Fournier (2016). "Effect of Implicit Perceptual-Motor Training on Decision-Making Skills and Underpinning Gaze Behavior in Combat Athletes." Perceptual &amp; Motor Skills **123**(1): 300-323.

Murgia, M., F. Sors, A. F. Muroni, I. Santoro, V. Prpic, A. Galmonte and T. Agostini (2014). "Using perceptual home-training to improve anticipation skills of soccer goalkeepers." Psychology of sport and exercise **15**(6): 642-648.

Nimmerichter, A., N. J. R. Weber, K. Wirth and A. Haller (2015). "Effects of Video-Based Visual Training on Decision-Making and Reactive Agility in Adolescent Football Players." Sports (Basel, Switzerland) **4**(1).

Paul, M., S. K. Biswas and J. S. Sandhu (2011). "ROLE OF SPORTS VISION AND EYE HAND COORDINATION TRAINING IN PERFORMANCE OF TABLE TENNIS PLAYERS." Brazilian Journal of Biomotricity **5**: 106-116.

Petri, K., S. Masik, M. Danneberg, P. Emmermacher and K. Witte (2019). "Possibilities to Use a Virtual Opponent for Enhancements of Reactions and Perception of Young Karate Athletes." International Journal of Computer Science in Sport (Sciendo) **18**(2): 20-33.

Phillips, J., M. Dusseault, T. Andre, H. Nelson and S. Polly Da Costa Valladão (2023). "Test Transferability of 3D-MOT Training on Soccer Specific Parameters." Research Directs in Strength and Performance **3**(1).

Rodrigues, P., J. Woodburn, A. J. Bond, A. Stockman and J. Vera (2025). "Light-based manipulation of visual processing speed during soccer-specific training has a positive impact on visual and visuomotor abilities in professional soccer players." Ophthalmic and Physiological Optics **45**(2): 504-513.

Romeas, T., R. Chaumillon, D. Labbe and J. Faubert (2019). "Combining 3D-MOT With Sport Decision-Making for Perceptual-Cognitive Training in Virtual Reality." Perceptual and motor skills **126**(5): 922-948.

Romeas, T., A. Guldner and J. Faubert (2016). "3D-Multiple Object Tracking training task improves passing decision-making accuracy in soccer players." Psychology of sport and exercise **22**: 1-9.

Schumacher, N., R. Reer and K. M. Braumann (2020). "On-Field Perceptual-Cognitive Training Improves Peripheral Reaction in Soccer: A Controlled Trial." Front Psychol **11**: 1948.

Schwab, S. and D. Memmert (2012). "The impact of a sports vision training program in youth field hockey players." Journal of Sports Science &amp; Medicine **11**(4): 624-631.

Serpell, B. G., W. B. Young and M. Ford (2011). "Are the perceptual and decision-making components of agility trainable? A preliminary investigation." J Strength Cond Res **25**(5): 1240-1248.

Theofilou, G., I. Ladakis, C. Mavroidi, V. Kilintzis, T. Mirachtsis, I. Chouvarda and E. Kouidi (2022). "The Effects of a Visual Stimuli Training Program on Reaction Time, Cognitive Function, and Fitness in Young Soccer Players." Sensors **22**(17): 6680.

Zwierko, M., W. Jedziniak, M. Popowczak and A. Rokita (2023). "Effects of in-situ stroboscopic training on visual, visuomotor and reactive agility in youth volleyball players." PeerJ **11**.
